# Spinal Myeloid Sarcoma Revealing Acute Myeloid Leukemia in a Child: A Case Report

**DOI:** 10.7759/cureus.113080

**Published:** 2026-07-21

**Authors:** Manal Azizi, Hasne Tkak, Anass Abderrahmani, Anass Haloui, Amal Bennani, Rachid Seddik, Ayad Ghanam

**Affiliations:** 1 Department of Pediatric Hematology-Oncology, Regional Oncology Center, Mohammed VI University Hospital/Faculty of Medicine and Pharmacy of Oujda (FMPO), Mohammed 1st University, Oujda, MAR; 2 Department of Pediatric Hematology-Oncology, Mohammed VI University Hospital/Faculty of Medicine and Pharmacy of Oujda (FMPO), Mohammed 1st University, Oujda, MAR; 3 Department of Pathology, Mohammed VI University Hospital/Faculty of Medicine and Pharmacy of Oujda (FMPO), Mohammed 1st University, Oujda, MAR; 4 Hematology Laboratory, Mohammed VI University Hospital/Faculty of Medicine and Pharmacy of Oujda (FMPO), Mohammed 1st University, Oujda, MAR

**Keywords:** acute myeloid leukemia, chloroma, extramedullary localization, myeloid sarcoma, spinal cord compression

## Abstract

Myeloid sarcoma (MS), formerly known as chloroma or granulocytic sarcoma, is a rare extramedullary malignant tumor composed of immature myeloid precursor cells. It is most commonly associated with acute myeloid leukemia (AML), although it may also occur in the setting of myelodysplastic syndromes or chronic myeloid leukemia, and occasionally precedes the diagnosis of systemic hematologic disease. Owing to its diverse clinical manifestations and non-specific radiological findings, diagnosis remains challenging and is frequently delayed. We report the case of an eight-year-old boy with no significant medical history who presented with a two-week history of dorsal back pain, asthenia, and progressive weakness of the lower limbs, followed by acute urinary and fecal incontinence. Clinical examination revealed acute flaccid paraplegia associated with thoracic spinal tenderness. Spinal magnetic resonance imaging (MRI) demonstrated an epidural mass causing spinal cord compression. Histopathological and immunohistochemical analyses confirmed the diagnosis of myeloid sarcoma. Further investigations established the diagnosis of AML-M2. The patient received systemic chemotherapy according to an AML treatment protocol, resulting in favorable neurological and clinical improvement.

## Introduction

Myeloid sarcoma (MS), also known as chloroma, is a rare extramedullary tumor composed of immature myeloid precursor cells, most commonly associated with acute myeloid leukemia (AML). It may occur concurrently with bone marrow involvement, during disease relapse, or, less frequently, precede overt hematologic manifestations and represent the initial presentation of AML [1,2].

In pediatric patients, epidural spinal involvement is exceptionally rare but constitutes a medical emergency because of the risk of spinal cord compression and irreversible neurological deficits. The clinical and radiological features are often nonspecific, making diagnosis challenging and potentially delaying appropriate treatment. Diagnosis relies on magnetic resonance imaging (MRI), while definitive confirmation requires histopathological examination supported by immunohistochemical analysis [3,4].

We report a pediatric case of spinal MS presenting as acute spinal cord compression that led to the diagnosis of underlying AML, highlighting the diagnostic challenges and therapeutic management of this uncommon presentation.

## Case presentation

A young boy aged eight years and nine months, the second of three siblings born to non-consanguineous parents, with no significant past medical history, presented with a two-week history of dorsal back pain, asthenia, and progressive lower limb weakness. Two days before admission, his condition worsened abruptly with urinary and fecal incontinence.

On physical examination, the patient was conscious and hemodynamically stable, with a heart rate of 100 beats per minute, blood pressure of 110/57 mmHg, oxygen saturation of 99% on room air, and no fever. Mild conjunctival pallor was noted. Neurological examination revealed acute flaccid paraplegia of both lower limbs, with complete motor deficit (Medical Research Council (MRC) grade 0/5), severe muscle hypotonia, absent patellar and Achilles tendon reflexes, and inability to stand or walk. A positive Bell sign with paravertebral muscle contracture was noted over the dorsal spine. The sensory level was not documented at the time of the initial neurological examination. No peripheral lymphadenopathy, hepatomegaly, splenomegaly, or hemorrhagic manifestations were observed.

**Figure 1:**
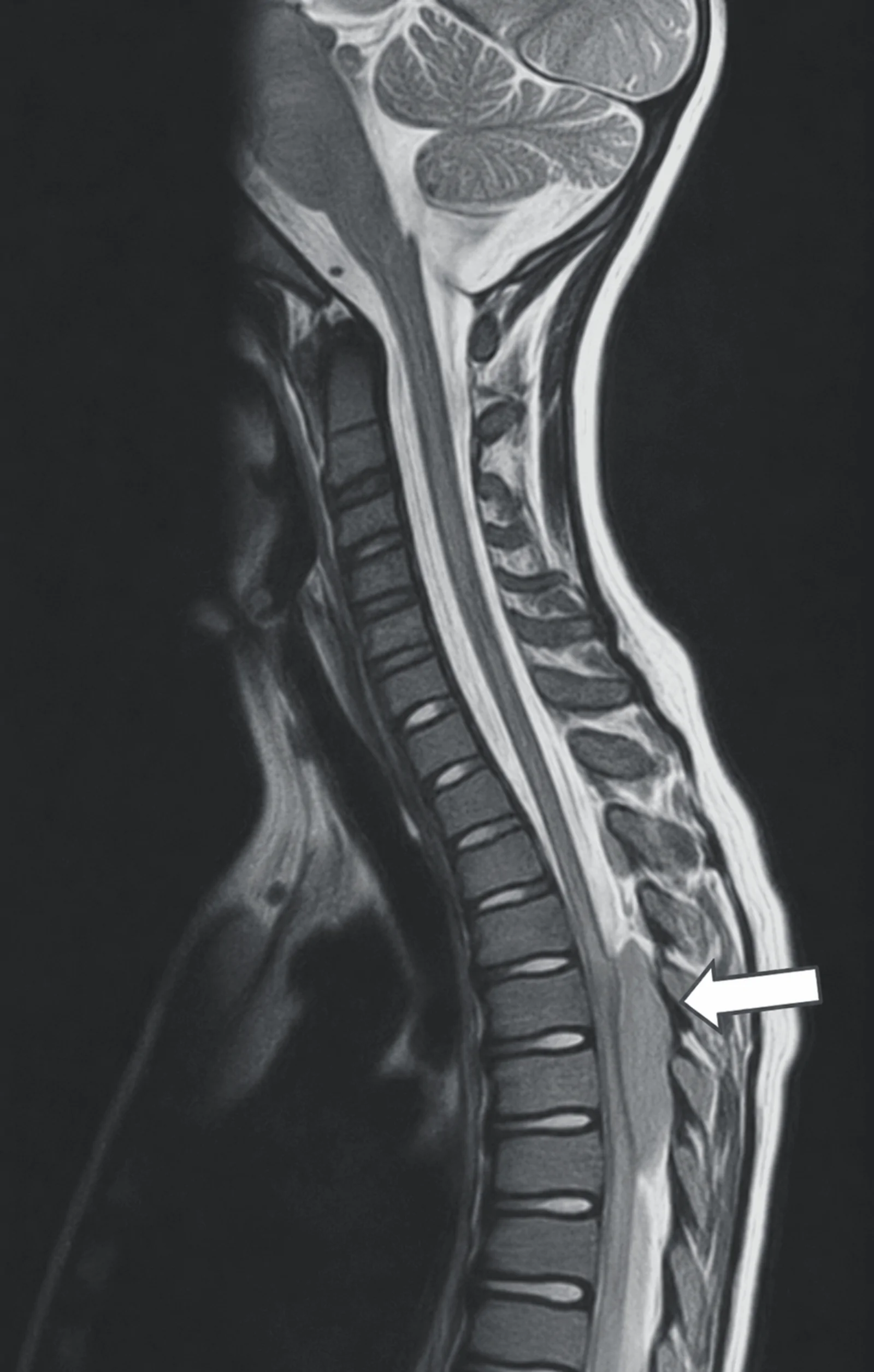
Preoperative sagittal T2-weighted spinal MRI showing a well-defined posterior epidural mass measuring 12 × 9 × 45 mm and extending from D4 to D6 (white arrow). The lesion appears hyperintense on T2-weighted images and is causing severe spinal cord compression with associated intramedullary T2 hyperintensity, consistent with secondary spinal cord edema due to mass effect. It also extends into the bilateral D4–D5 and D5–D6 neural foramina and is in close contact with the posterior vertebral arches, without evidence of vertebral body involvement.

Urgent spinal MRI demonstrated a posterior epidural mass extending from D4 to D6, isointense on T1-weighted images and hyperintense on T2-weighted images, causing severe spinal cord compression with associated spinal cord edema (Figure 1).

Based on the clinical presentation and imaging findings, the main preoperative differential diagnoses included lymphoma, neuroblastoma metastasis, Ewing sarcoma, epidural abscess, and other small round blue cell tumors. Laboratory investigations are summarized in Table 1.

**Table 1:** Laboratory findings at diagnosis. LDH: lactate dehydrogenase

Parameter	Patient value	Reference range	Interpretation
Hemoglobin	6.9 g/dL	11–14 g/dL	Decreased
White blood cell count	12.41 ×10³/µL	4.5–13.5 ×10³/µL	Normal
Neutrophils	26% (3.23 ×10³/µL)	30–70% (1.5–8.0 ×10³/µL)	Relative neutropenia
Platelets	33 ×10³/µL	150–400 ×10³/µL	Decreased
Peripheral blasts	24%	0%	Increased
Bone marrow blasts	39%	<5%	Increased
LDH	1755 U/L	120–300 U/L	Increased
Uric acid	52 mg/L	20–55 mg/L	Normal
C-reactive protein (CRP)	14 mg/L	<5 mg/L	Increased
Prothrombin time (PT)	100%	70–100%	Normal
Fibrinogen	2.6 g/L	2.0–4.0 g/L	Normal

**Figure 2:**
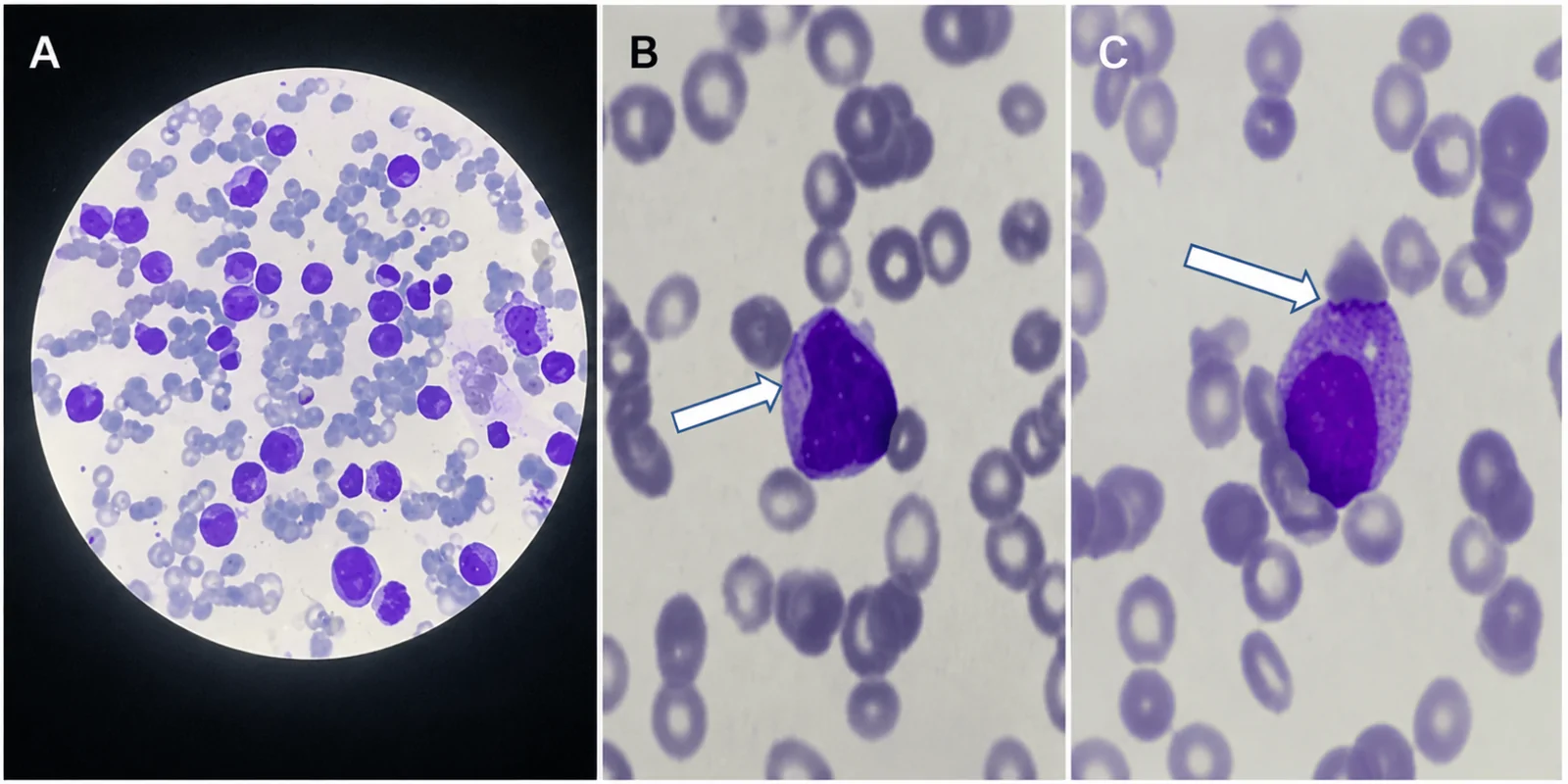
Bone marrow smear showing myeloblast infiltration with azurophilic granulations and Auer rods, consistent with acute myeloid leukemia. A: Circulating blasts at 24% with the presence of azurophilic granulations and Auer rods, consistent with acute myeloid leukemia. B and C : A blast cell containing an Auer rod (white arrows).

Peripheral blood smear demonstrated circulating blasts with myeloid morphology, including Auer rods. Bone marrow examination confirmed blast infiltration consistent with AML (Figure 2).

Immunophenotyping revealed blasts partially expressing CD34, HLA-DR, CD117, CD33, CD13, and CD15, with intracellular myeloperoxidase positivity and absence of T- or B-cell lineage markers. Conventional cytogenetic analysis demonstrated a normal karyotype. Additional molecular genetic testing was not available and therefore could not be performed. Overall findings were consistent with AML-M2 according to the French-American-British (FAB) classification [5].

Intravenous corticosteroid therapy was initiated immediately after the diagnosis of acute spinal cord compression to reduce spinal cord edema and preserve neurological function. Given the severity of the neurological impairment, the patient subsequently underwent emergency neurosurgical decompression consisting of a D4-D5 laminectomy with partial extension to D6 and partial resection of the extradural mass. Intraoperatively, the lesion appeared infiltrative and firmly adherent to the dura mater.

**Figure 3:**
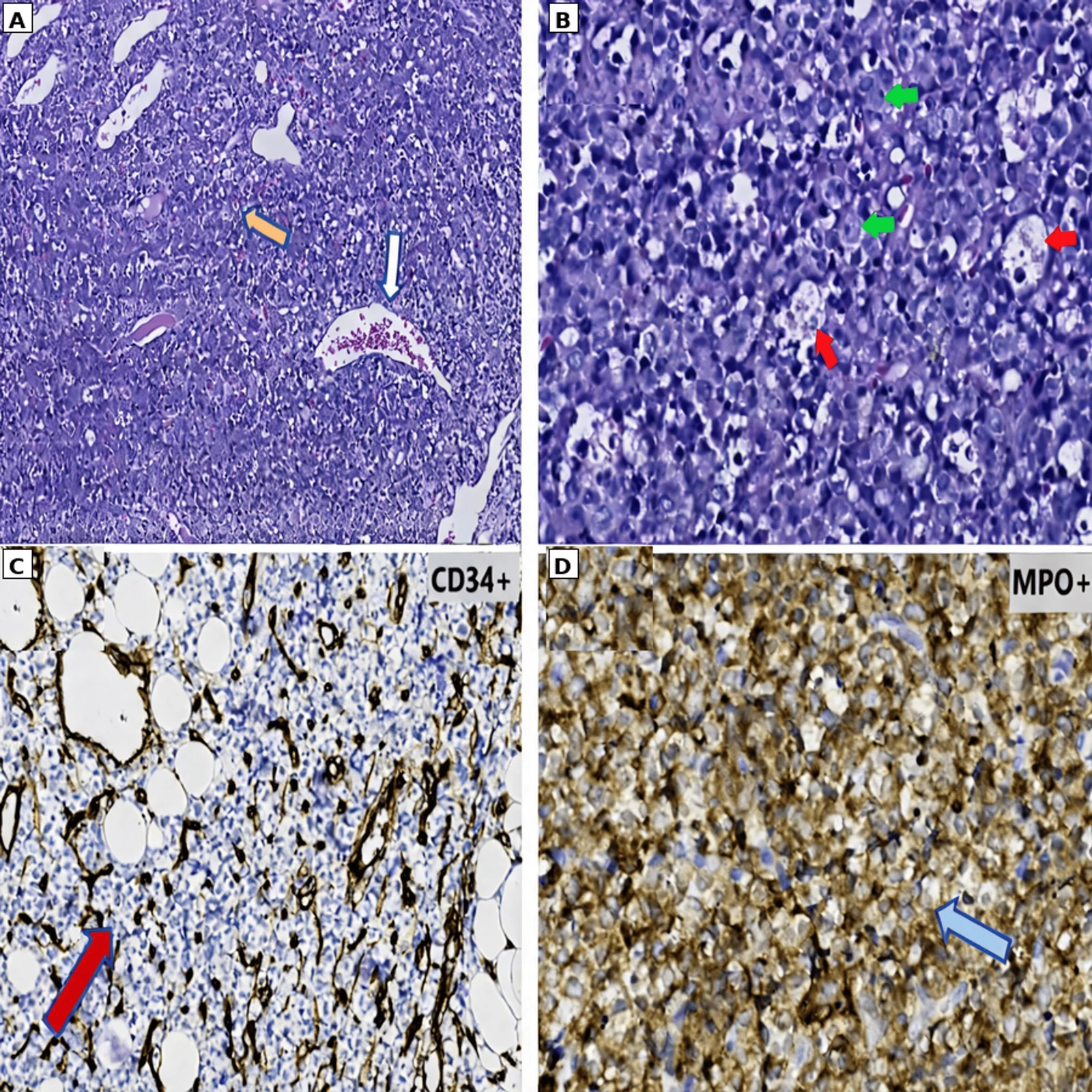
Histopathological examination of the surgical specimen A: Medium power view  showing a tumoral proliferation forming cohesive sheets and nests set within a fibro-inflammatory stroma (orange arrow) with numerous dilated vascular channels (white arrow) (Hematoxylin-Eosin-Saffron (HES) stain, magnification x 10). B: High power view demonstrating immature blastic cells provided with round or ovoid nuclei, fine chromatin with prominent nucleoli and a slightly basophilic cytoplasm with indistinct boundaries (green arrow) . Mitotic figure and numerous tangible body macrophages (red arrow) with apoptotic bodies are seen (HES stain, magnification x40). C: CD34 stained scattered tumor cells (red arrow). D: Tumor cells showing diffuse granular cytoplasmic staining  of myeloperoxydase (blue arrow).

Histopathological examination of the surgical specimen confirmed MS as an extramedullary manifestation of AML (Figure 3).

**Figure 4:**
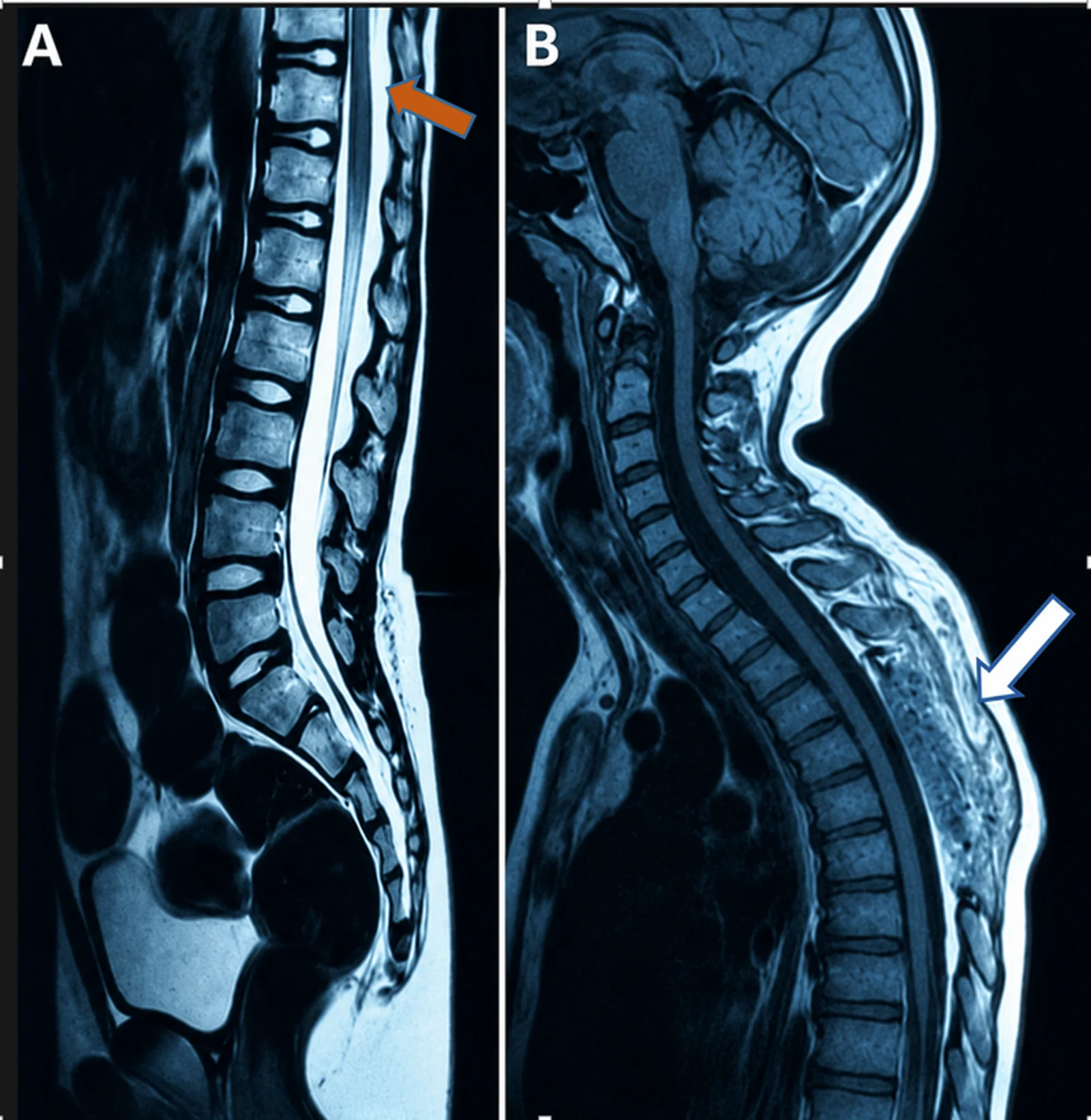
Postoperative spinal MRI A: T2-weighted thoracic spinal MRI showing complete disappearance of the posterior epidural lesion (brown arrow) after laminectomy, with resolution of spinal cord compression. B: Sagittal STIR thoracic spinal MRI demonstrating persistent intramedullary hypersignal at D4–D6 levels (white arrow), consistent with residual spinal cord injury. STIR: short TI inversion recovery

Postoperative spinal MRI demonstrated complete removal of the epidural mass with resolution of spinal cord compression. Persistent intramedullary T2 hypersignal extending from D4 to D6 suggested residual spinal cord edema secondary to the previous compression (Figure 4).

The patient subsequently received spinal radiotherapy for local disease control at a total dose of 30 Gy delivered in 15 fractions, followed by induction chemotherapy according to the national AML-MA-2011 protocol [6].

Induction therapy was completed successfully, and bone marrow evaluation at the end of induction showed 3% blasts, consistent with hematological remission. After six months of follow-up, the patient is receiving the third consolidation cycle. Neurological recovery was favorable, with complete restoration of sphincter function, recovery of muscle strength to grade 5/5 in both lower limbs, and the ability to ambulate with assistance.

## Discussion

MS is an extramedullary tumor composed of immature myeloid precursor cells and is recognized by the World Health Organization (WHO) as a distinct pathological entity [7,8]. It presents as a localized mass of leukemic cells occurring outside the bone marrow.

MS develops in approximately 10-15% of patients with AML and may also be associated with myelodysplastic syndromes or chronic myeloid leukemia [9]. In nearly 25% of cases, it arises in the absence of a previously diagnosed hematologic disorder; however, most patients subsequently develop AML within 6-10 months [10,11]. In the present case, spinal MS represented the inaugural manifestation of AML, making the diagnosis particularly challenging and highlighting the importance of considering this entity in children presenting with spinal cord compression of unclear etiology.

The condition is more common in children than in adults, with a slight male predominance, and nearly 60% of reported cases occur before the age of 15 years [2,4,12]. The most frequently affected sites include bone, skin, and lymph nodes, whereas spinal involvement remains rare and predominantly affects the thoracic region [3,13,14]. Epidural involvement can lead to acute spinal cord compression, which may be the first manifestation of AML. Consistent with these observations, our patient presented with a dorsal epidural mass causing severe spinal cord compression.

Clinical presentation varies from localized pain and progressive neurological deficits to acute paraplegia. Misdiagnosis rates may reach up to 50%, particularly in cases with isolated spinal involvement because of the rarity and nonspecific presentation of the disease [15]. Our patient developed paraplegia associated with sphincter dysfunction, reflecting the severity of neurological compromise reported in similar pediatric cases.

MRI remains the imaging modality of choice for evaluating spinal lesions. Typically, MS appears as an epidural mass that is isointense relative to muscle on T1-weighted sequences and intermediate-to-hyperintense on T2-weighted sequences [14,16]. MRI also plays a crucial role in assessing the extent of disease and the degree of spinal cord compression. In our patient, MRI demonstrated significant spinal cord compression, supporting the indication for urgent surgical intervention. These findings highlight the importance of MRI in guiding both diagnosis and therapeutic decision-making in cases of spinal myeloid sarcoma. Nevertheless, radiological diagnosis remains challenging because imaging findings are nonspecific and may mimic neoplastic or inflammatory soft-tissue lesions [14,15]. Consequently, definitive diagnosis relies on histopathological examination.

Histologically, MS demonstrates heterogeneous morphology ranging from well-differentiated lesions to undifferentiated blastic forms composed of immature non-granular myeloblasts, making diagnosis difficult. Immunohistochemistry plays a crucial diagnostic role, with frequent positivity for myeloperoxidase and lysozyme markers [17]. In our case, histopathological and immunohistochemical analyses were essential to establish the diagnosis and differentiate MS from other pediatric small round blue cell tumors. Certain cytogenetic and molecular abnormalities, particularly t(8;21), have been associated with an increased risk of MS development and may influence treatment response and prognosis [9,14]. However, molecular characterization was not available for our patient, representing a limitation of the present report.

Management of spinal MS relies on a multimodal therapeutic strategy combining systemic chemotherapy, local radiotherapy, and surgical intervention in selected cases. Chemotherapy regimens generally follow AML treatment protocols, including induction therapy with cytarabine and anthracyclines followed by consolidation therapy with high-dose cytarabine.

In cases of significant mass effect or acute spinal cord compression, urgent surgical decompression may be required to relieve neurological symptoms and obtain tissue for histopathological diagnosis. Accordingly, our patient underwent emergency decompressive laminectomy because of paraplegia and sphincter dysfunction. Given the severity of the neurological impairment and the persistence of spinal cord compression, radiotherapy was administered before the initiation of AML-directed chemotherapy to achieve rapid local disease control and minimize the risk of further neurological deterioration. Although MS is generally highly responsive to systemic chemotherapy, local treatment modalities such as surgery and radiotherapy may be necessary in selected patients with severe neurological compromise [18].

Recent studies indicate that patients with MS who receive early and appropriate treatment may achieve complete remission and substantial neurological recovery [4,12,19]. The favorable clinical, neurological, and hematological evolution observed in our patient supports these findings. These encouraging results emphasize the importance of early diagnosis and rapid initiation of multidisciplinary treatment. However, relapse remains possible, and long-term follow-up is required. In refractory or recurrent disease, allogeneic hematopoietic stem cell transplantation may be considered to improve disease control and survival outcomes [20].

This case adds to the limited literature on pediatric spinal MS presenting as the inaugural manifestation of AML and highlights the favorable neurological and hematological outcomes that can be achieved through early recognition and prompt multimodal management.

## Conclusions

Spinal MS is a rare but severe extramedullary manifestation of AML in children and may represent its initial presentation. Diagnosis remains challenging because of nonspecific clinical and radiological findings, particularly in isolated or hematologically silent cases. MRI combined with histopathological and immunohistochemical examination is essential for establishing an accurate diagnosis. Early multidisciplinary management, including systemic chemotherapy, radiotherapy, and surgical decompression when indicated, is crucial to optimize neurological recovery. This case highlights the importance of considering MS in the differential diagnosis of pediatric spinal cord compression to facilitate timely diagnosis and appropriate treatment.
